# Protein–Protein Interaction Prediction for Targeted Protein Degradation

**DOI:** 10.3390/ijms23137033

**Published:** 2022-06-24

**Authors:** Oliver Orasch, Noah Weber, Michael Müller, Amir Amanzadi, Chiara Gasbarri, Christopher Trummer

**Affiliations:** Celeris Therapeutics GmbH, Salzamtsgasse 7, 8010 Graz, Austria; o.orasch@celeristx.com (O.O.); n.weber@celeristx.com (N.W.); m.mueller@celeristx.com (M.M.); a.amanzadi@celeristx.com (A.A.); c.gasbarri@celeristx.com (C.G.)

**Keywords:** protein–protein interactions, targeted protein degradation, ternary complex, deep graph representation learning

## Abstract

Protein–protein interactions (PPIs) play a fundamental role in various biological functions; thus, detecting PPI sites is essential for understanding diseases and developing new drugs. PPI prediction is of particular relevance for the development of drugs employing targeted protein degradation, as their efficacy relies on the formation of a stable ternary complex involving two proteins. However, experimental methods to detect PPI sites are both costly and time-intensive. In recent years, machine learning-based methods have been developed as screening tools. While they are computationally more efficient than traditional docking methods and thus allow rapid execution, these tools have so far primarily been based on sequence information, and they are therefore limited in their ability to address spatial requirements. In addition, they have to date not been applied to targeted protein degradation. Here, we present a new deep learning architecture based on the concept of graph representation learning that can predict interaction sites and interactions of proteins based on their surface representations. We demonstrate that our model reaches state-of-the-art performance using AUROC scores on the established MaSIF dataset. We furthermore introduce a new dataset with more diverse protein interactions and show that our model generalizes well to this new data. These generalization capabilities allow our model to predict the PPIs relevant for targeted protein degradation, which we show by demonstrating the high accuracy of our model for PPI prediction on the available ternary complex data. Our results suggest that PPI prediction models can be a valuable tool for screening protein pairs while developing new drugs for targeted protein degradation.

## 1. Introduction

Tackling diseases is one of humankind’s most long-lasting challenges. An essential part of this effort concerns targeting pathogenic proteins (e.g., misfolded proteins in CNS diseases or mutated proteins in cancer). Since Fischer’s “Lock and Key” model [1], drug discovery has followed the occupancy-driven drug discovery paradigm by focusing on designing molecules capable of fitting the active site of the target protein. This binding leads to inhibition and subsequent loss of the protein of interest (POI) function. Although this approach has been successful in many cases, it is limited by the fact that less than 20% of the human proteome is inhibitable; i.e., most POIs do not contain binding sites that compounds can occupy [2,3,4]. To address this shortcoming and expand the druggable space, new therapeutic modalities such as gene knockout (CRISPR-Cas9) [5] and gene expression knockdown (RNAi) [6] have been developed. An alternative lies in proximity-inducing compounds (PICs), which retain the advantages of small molecules (e.g., oral bioavailability and ease of production, [7]). PICs allow inducing proximity between POIs and proteins driving various cellular processes [8] such as phosphorylation [9], dephosphorylation [10], deacetylation [11] and protein degradation [12], allowing to repurpose the elaborate machinery available in every cell.

**Figure 1:**
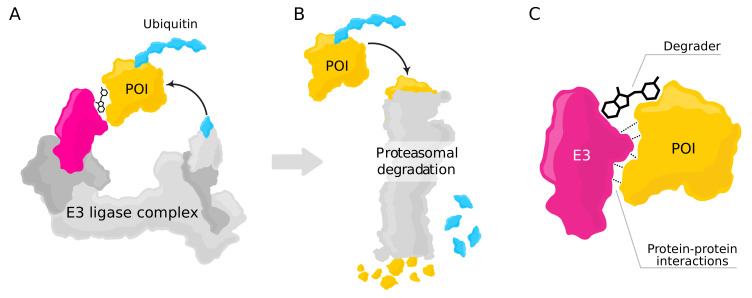
The relevance of protein–protein interactions for targeted protein degradation with bifunctional degraders. (**A**) To accomplish targeted protein degradation, the protein of interest (POI, yellow) is linked to the receptor (magenta) of an E3 ligase complex via a degrader molecule. Together, the POI, degrader, and E3 ligase form a ternary complex, allowing the passage of ubiquitin to the POI. (**B**) Ubiquitination of the POI leads to its degradation via the proteasomal system. The ubiquitin is recycled. (**C**) While the degrader molecule is instrumental for bringing the two proteins into proximity, cooperativity between the E3 ligase and the POI—i.e., strong protein–protein interactions—is essential for the formation of a stable ternary complex.

One mode of PIC-based drug design is targeted protein degradation (TPD) with bifunctional degraders, which was first described in the early 2000s [12]. The degraders used for TPD are small molecules that induce proximity between a POI and the E3 ligase complex (Figure 1A). This proximity allows the passage of ubiquitin from the E3 ligase complex onto the POI, triggering its subsequent degradation by the ubiquitin–proteasomal system (Figure 1B; see [13]). TPD, therefore, results in the selective degradation of proteins, which is accomplished by hijacking the existing degradation pathways in human cells. This approach has several advantages [14,15] such as allowing to target proteins that are not inhibitable by conventional means, and thus, it has become a significant research focus within the field of drug discovery [15,16,17].

The development of new drugs operating with TPD requires predicting the interactions between the E3 ligase and the POI. Strong protein–protein interactions (PPIs) are required for the formation of a ternary complex (E3 ligase–degrader–POI, see Figure 1C), which is a prerequisite for POI ubiquitination [18]. If the two proteins do not interact favorably, the degrader molecule will not induce the formation of a ternary complex. In this case, for a particular POI, a different E3 ligase with favorable interactions might be considered instead (commonly used E3 ligases include, among others, CRBN and VHL [19]) if reliable PPI prediction methods are available.

There are different approaches to studying and predicting PPIs [20]. Experimental techniques are reliable for detecting structures of small proteins or single monomers, but they have intrinsic limits regarding protein complexes (see, e.g., [21,22]) and are time- and resource-intensive [23]. To overcome these limitations, computational methods to predict 3D protein complexes and the corresponding PPIs have been developed. Currently available methods, many of which rely on machine learning, can be classified into two categories: template-free (“ab initio”) and template-based approaches [23].

The ab initio docking methodology allows the predictive discrimination of cognate vs. non-interacting protein candidates without the requirement for experimental data. The appropriate matching of physicochemical properties, as well as the geometrical complementarity of the candidates’ potential binding sites, are crucial criteria for success in the determination of productive protein–protein interactions. While computationally expensive, the template-free approach can nevertheless be a valuable tool in the modeling of PPI due to its accuracy, exclusion of experimental bias and in scenarios where either experimental data are scarce or uncovering novel modes of interactions is of interest [24,25].

Homology or template based docking approaches (e.g., [26]) rely on experimentally elucidated structures of protein–protein complexes characterized by a significant degree of similarity to the target proteins [27]. Therefore, the initial stage of the docking protocol is reduced from an extensive global search of potentially interacting candidates (as within the methodology described above) to a structural alignment of the binding sites in question to the template selection, resulting in a significant reduction of computational cost. Here, success in the accurate prediction of PPIs is crucially dependent on the diversity/similarity and the size of the template dataset considered, providing the additional advantage of a highly probable implicit inclusion of relevant conformational orientations of the residues within the binding sites, favorably affecting the computation time for the refinement step [28]. Unfortunately, these data are generally scarce [27], in particular for ternary complexes in the context of TPD (where at the time of this writing, only sixteen structures were available in the Protein Data Bank [29]).

This work presents a new framework for PPI prediction and shows its usefulness for predicting the interactions within the available ternary complex data. Our model processes proteins via their surface mesh [30] using deep graph representation learning (DGRL, see [31]), which is a popular approach of processing graph-based information such as molecular structures that has been shown to result in superior models within the field of computational drug discovery [32]. We overcome the problem of limited training data by employing transfer learning [33]: for training our model, we use data from ordinary PPIs (available at a larger scale) and use the trained model to predict the PPIs within ternary complexes. We show that our model is competitive with the current state-of-the-art template-based PPI prediction method [26]. We furthermore introduce a new PPI dataset including more diverse data than the commonly used MaSIF dataset [30], and we show that it is essential for capturing the broad range of PPIs present in ternary complexes. Our results suggest that PPI prediction can predict the PPIs underlying new ternary complexes and thus may serve as an essential tool in the development of new drugs for TPD.

## 2. Methods

**Figure 2:**
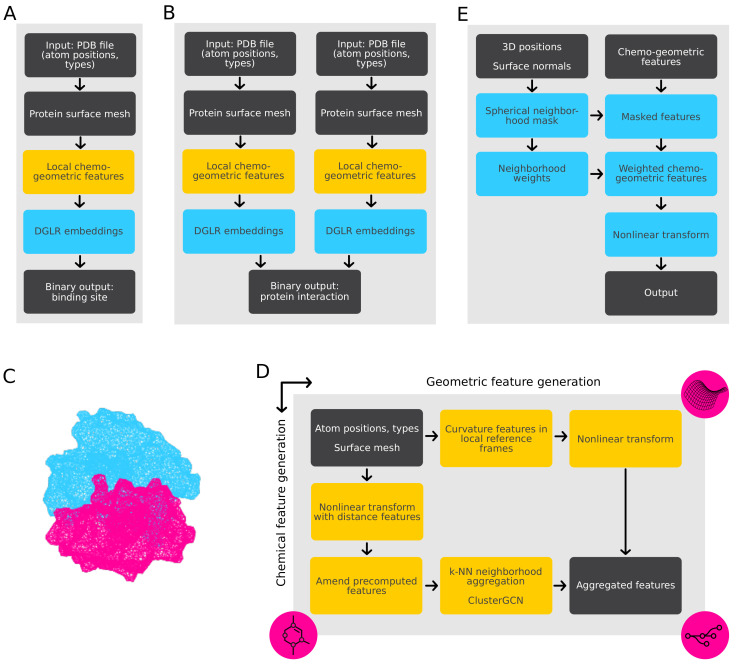
Model details. (**A**) Overall workflow for binding site prediction. The model outputs binary predictions of site activity for different points on the surface of proteins which are input via PDB files. The main processing steps are surface mesh generation followed by the computation of local chemo-geometric features, which are processed in a DGRL pipeline. (**B**) Overall workflow for interaction prediction of two proteins. Each protein is processed separatedly in a pipeline similar to that used for binding site prediction. The final processing step combines the learned features and produces a binary output. (**C**) Example of meshed protein surfaces of hemoglobin alpha and beta chains (PDB ID: 1A01, computed with EDTSurf [34,35]). Our strategy toward fines of the mesh was finding a sweet spot between rough meshes and fine meshes, where we would have a detailed representation of the surface while allowing for fast operations. Default parameters from the EDTSurf software were taken, where the probe radius is set to 1.4 as described in the EDTSurf documentation. (**D**) Details of chemo-geometric feature generation. Chemical and geometrical features are generated in separate streams. The geometrical feature generation (horizontal) consists of a learned embedding of curvature features estimated in the neighborhood of a point under consideration (see text for details). The chemical feature computation (vertical) includes learned embeddings with added distance-dependent and precomputed chemical features, which are aggregated within a neighborhood and processed with a single elementary Cluster-GCN layer [36,37] (see text for details). (**E**) Details of final DGRL processing. The chemo-geometric within a spherical neighborhood around a point under consideration influences the features at this point using learned weights generated from 3D positions and surface normals. The weighted features are processed with a multilayer perceptron (MLP), resulting in the final output.

This section describes the details of our model architectures, which map 3D atomic protein structures from the Protein Data Bank (PDB [29]) to binary outputs. We consider two PPI prediction tasks (see [26]):

- Binding site prediction: One PDB file is used as model input, and the binary output describes whether a particular location on the protein surface constitutes a possible site for protein interactions (see Figure 2A).
- Interaction prediction: Two PDB files are processed, and the binary output describes whether the two proteins interact at a particular site (see Figure 2B).

In both cases, outputs are generated for various locations on the protein surface. Since our models for these two tasks share most of their components, we first provide a high-level overview before describing the components in detail.

### 2.1. Model Overview

The overall model architectures for both the prediction of interaction sites (Figure 2A) and protein interaction prediction (Figure 2B) are very similar, as in both cases, the input consists of proteins and the output consists of a binary value. In particular, the initial processing sequence of protein surfaces estimation, chemo-geometric feature generation, and processing with DGRL are identical. The differences lie in the final processing layers, where we learn the binary classification specific to one of the two tasks: interaction site prediction (requiring input from only a single protein) or the prediction of PPIs (requiring two inputs). Additionally, the weights of the trainable components within the two pipelines are not identical and are trained separately for their respective tasks. We furthermore performed separate hyper-parameter tuning for the two different cases.

Our model uses a deep graph-representation learning (DGRL) pipeline to process the structural information of the proteins using graph representations. The input is based on surface meshes (Figure 2C), which are commonly considered to be suitable graph representations for proteins [30]. We use this representation together with domain-specific features (Figure 2D) and process them using graph-based convolutional neural networks (Figure 2E), which are engineered to respect the geometric and chemical properties of the biomolecules. In the graph-based representation, nodes within a neighborhood share common properties, thus allowing the model to reproduce real-world effects of local molecular interactions. Such relations, which are expressed with edges in the graph, can be “summarized” with the help of weight sharing. This allows our pipeline to learn expressive features that accurately discriminate protein–protein interactions.

### 2.2. (Pre-)Processing of 3D Structures into Graph Representations

The first processing step maps the initial 3D protein structures from PDB to a suitable surface representation. We use meshes created by EDTSurf [34,35], where the minimal macromolecular surface of the underlying atomic point cloud is computed using a Euclidean distance field. Hence, the relevant graph data structures are the virtual surfaces represented by the mesh vertices. The edges are generated on the fly when required (see below).

### 2.3. Chemo-Geometric Feature Generation

Our model learns expressive chemo-geometric features based on 3D atom coordinates, atom types, surface representation, and selected precomputed features (e.g., hydrophobicity) of the protein at each surface location under consideration. These features are fed to different neural network architecture and optimized to learn the most suitable chemical and geometric embeddings (Figure 2D). The results are concatenated and used as input to the DGRL model. In the following, we describe the details of chemical and geometric feature generation.

#### 2.3.1. Chemical Features

Since we are working with surface representations of proteins, it is necessary to aggregate the near-surface atomic information into abstract feature vectors assigned to the mesh vertices. We, therefore, embed the chemical features using a *k*-NN directed graph; i.e., for each surface point, we aggregate the features of the *k* nearest atoms. The chemical information associated with these atoms is processed via DGRL methods through an elementary convolution operator as described in [37]. As input, we provide the 3D coordinates of the atoms and the surface representation and atom types as a one-hot encoded vector. To study subtle differences between edge lengths across all proteins, we further add Fourier distance features [38] defined by

(1) $$\gamma \left(d\right)=(d,\cos\left(d/2^{0}\right),\;\sin\left(d/2^{0}\right),\;\ldots ,\;\cos\left(d/2^{F}\right),\;\sin\left(d/2^{F}\right)),$$

These are embedded together with the raw atomic information (i.e., nonlinearly transformed by an MLP). Although neural networks can learn complex features without feature engineering [39], we enhance this process by adding some essential features which are known to be relevant for the tasks: hydrophobicity and hydrogen bond potential. For this, we follow the preprocessing pipeline described in [30], where for every surface patch containing this raw atomic information, we determine and quantify these features.

#### 2.3.2. Geometric Features

An important feature that becomes available when assuming a sufficiently smooth surface is curvature. We assume that the shape of surface patches, quantified by curvature features, plays an essential role in identifying interaction sites (see, e.g., [40,41]). In the current literature, two approaches of incorporating curvature are prevalent. The first approach relies on the Gaussian [42] and mean curvatures [43], which are intrinsic and extrinsic measures of curvature, respectively. The second approach employs the so-called shape index and the curvedness, which reflect the local shape of a surface patch and problem-specific length scales [44]. Both methods depend on the principal curvatures $k_{1}$ and $k_{2}$ [45], i.e., minimal and maximal normal curvatures, which are the eigenvalues of the shape operator [46]

(2) $$S=-\;\nabla_{\vec{v}}\;\vec{N}$$

on the tangent basis. Intuitively, *S* measures the deviation of the normal vector field $\vec{N}$ with respect to an arbitrary tangent vector $\vec{v}$. As is typical when dealing with meshes, the normal vertex vectors are estimated by averaging over the neighboring face normal vectors, which are computed using standard trigonometric procedures from the edges of the mesh.

To compute *S*, we employ the strategy introduced in [47]. We pick *k* nearest neighbors of a mesh node and project the relative vectors and the differences of the normal vectors into its tangent plane. First, a local basis needs to be constructed from the normals. Subsequently, we project the relative coordinates and relative normals to the tangent plane, and the shape operator *S* can be estimated.

In contrast to the two approaches described above, we do not rely on the principal curvatures’ explicit function(s). However, we do not use the raw geometric information imprinted in the principal curvatures, since these quantities are coordinate-dependent. Thus, we diagonalize *S* and provide the eigenvalues, i.e., the principal curvatures $k_{1}$ and $k_{2}$, as input to a shallow MLP, which learns a latent representation suitable for downstream use.

### 2.4. Main DGRL Pipeline

The main DGRL [31] pipeline processes the chemo-geometric embeddings using the 3D coordinates and surface normals as additional input (Figure 2E). It performs nonlinear geometry-informed processing of the features, resulting in a binary classification output. The underlying concept of this pipeline is weight sharing, i.e., convolutions. Most properties of an atom or surface point depend on its immediate neighborhood. Assuming that points in a neighborhood interact similarly across different neighborhoods, the choice of convolutional layers is natural to calculate the low-dimensional representations of these properties. While our focus is the classification of binding sites and PPIs, this pipeline could also be used to learn other target values (e.g., a docking score).

The input of this processing step consists of the 3D coordinates and surfaces normals of the surface points under consideration, as well as the chemo-geometric features computed at those points. At each location, the features of all nearby points within a fixed radius are weighted and aggregated (Figure 2E). For a particular location, the weights describe the influence of all positions within the considered radius and are computed by an MLP, which maps the differences of point positions and surface normals to a scalar. As the MLP is applied to all possible point pairings, this operation step corresponds to a spatial convolution. The weights are then applied to the chemo-geometric features (transformed by an MLP), which are aggregated at each point by summation over the weighted features of all neighboring points. The diameter of the sphere results in O(100–1000) neighbors per query point; thus, there are thousands of pairs per protein molecule, resulting in prohibitively large memory consumption if conventional methods are used. This problem is circumvented through the use of processing with symbolic operations, as provided by the PyKeOps library [48].

Finally, the aggregated features at each point are processed by a final MLP, resulting in an eight-dimensional feature vector per point, which is mapped to a binary output in the case of binding site prediction. For the interaction prediction task (Figure 2B), the outputs of the two proteins are further processed by another MLP, mapping to a binary output.

### 2.5. Model Training

To label the surface meshes into binding and non-binding vertices and aggregate the evaluation metric of the predictions, we followed a similar strategy as described in [30]. For interaction site prediction, all possible interaction sites were registered. For PPI prediction, we used an equal number of positive labels (i.e., surface patches on both proteins where they interact) and negative labels (i.e., surface patches on both proteins where they do not interact) to ensure a balanced dataset. The number of these discrete surfaces is determined by the size of the *k*-NN ball query.

We trained our models (i.e., the trainable parameters of the various MLPs, the ClusterGCN layer, etc.) using stochastic gradient descend with the AMSGrad version of the Adam optimizer [49]. We used the binary cross-entropy between the model output and the true target values as a loss function. To avoid overfitting, we employed an early stopping strategy [39], for which the model was evaluated on a validation set (10% of the training set) during training.

**Figure 3:**
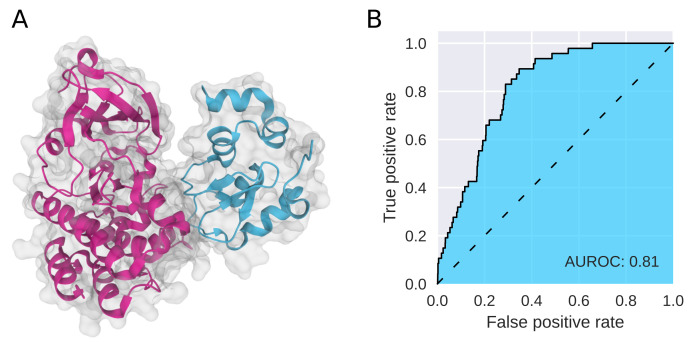
Example evaluation on ternary complex data. (**A**) PPIs of proteins involved in the ternary complex composed by BTK, cIAP ubiquitin ligase and compound 17 (PDB ID: 6W7O). (**B**) Corresponding AUROC curve. See Appendix B for predictions on additional ternary complex data.

To evaluate our models, we followed [26] and used the AUROC (area under the receiver operating characteristic curve [50]) as an evaluation metric. The final AUROC value summarizes predictions across all surface patches and quantifies the ratio of true positive rate vs. true negative rate for different cut-off values. For an example ternary complex (6W7O, Figure 3A), the AUROC is visualized in Figure 3B. As the final AUROC score for a protein–protein pair includes averaging over multiple positive and negative surface patch predictions involved in the PPIs, a high AUROC score for a single protein–protein interaction means that the model can accurately predict whether proteins will interact based on the predictive power on individual surface patches of the two proteins.

### 2.6. Implementation

The model described above was implemented using the libraries PyTorch [51], PyTorchGeometric [36], and PyKeOps [48].

## 3. Results

We present in this work a model capable of predicting possible interaction sites and whether two proteins will interact. As our goal is to perform these prediction tasks in the context of targeted protein degradation, we emphasize achieving good results on existing data of ternary complexes (consisting of two proteins and a degrader molecule). We first present the results of our model on two protein–protein interaction (PPI) datasets before discussing this particular scenario.

### 3.1. The Orthogonal Dataset

To train and evaluate our model, we use two datasets. The first one, commonly called the MaSIF dataset, was used to evaluate the MaSIF model [30], which is an existing approach for PPI prediction, and it consists of protein pairs from several different sources, including PDB [30]. As implemented in the MaSIF source code, in preprocessing, the MaSIF utilizes a distance cut-off value of 1.4 Å to assess if two surface points on different side chains are interacting or not. Since the model employs surface point clouds representation as inputs, we reasoned that having protein structures with higher crystallographic resolution could lead to more precise predictions, especially for identifying interaction sites. Furthermore, a more diverse dataset allows testing the generalizability of PPI prediction approaches, resulting in more robust results. We thus generated a new dataset by selecting all Homo sapiens proteins within PDB with a resolution of less than or equal to 2 Å, which were not included in the MaSIF dataset. The protein pairs in the resulting dataset, which we call Orthogonal, have an average resolution of 1.7 Å (see Table 1).

We processed the selected protein’s FASTA sequences to the InterPro software [52], which categorized their structures into protein superfamilies and assigned a similarity score to each protein. The similarity scores were used to generate training and testing datasets that included a balanced representation of all protein classes and families. The main reason for the sequencing analysis is that we aim to obtain as much structural variability as possible to make the final model less susceptible to out-of-distribution protein structures.

It was necessary to visually examine all of the PDB structures in the Orthogonal dataset to determine which chains of each PDB structure are represented in a binary protein–protein interaction. We selected two chains for each PDB ID that reflects the PPI based on the proximity of the chains’ 3D structures. Based solely on visual examination, we also speculated that the proteins in the orthogonal dataset had a higher number of tertiary protein structures than MaSIF, which could assist the model in being more generalizable and tolerating to surface variance of the proteins. This property is critical because tertiary protein structures are found in practically all highly complex biological systems, including transcription factors, signaling proteins, and membrane proteins, all of which are critical for drug discovery and development.

In total, the Orthogonal dataset contains 2373 and 1111 distinct PDB IDs for training and testing our model of binding site prediction, respectively, and 3201 and 1431 PDB IDs for training and testing of the protein–protein interaction predictions. As the ratio of test data to training data is very high, the evaluation results on the test set of this dataset are likely to be a more accurate and reliable indicator of the generalization capabilities of a model compared to the dMaSIF dataset (where the ratio is lower). Furthermore, the dataset also includes rare complex protein residues that have heavy atoms bound to the side chain of the amino acids and operate as an organometallic bridge, providing more diverse data for further improvement of the evaluation accuracy.

### 3.2. PPI Prediction on Protein Pairs

We trained and evaluated our model (see Section 2) using these two datasets and compared it to two prominent PPI prediction models (Table 2): MaSIF and dMaSIF (note that MaSIF refers to both a model and a dataset). For the MaSIF dataset, our model shows better results on the interaction prediction task, while dMaSIF results in higher accuracy on the binding site prediction task. While the former is arguably more critical for our goal of predicting PPIs for targeted protein degradation, we overall found no significant improvement on this dataset. Conversely, when evaluating the models on the Orthogonal dataset, we find that our model clearly outperforms dMaSIF (Table 2). For the task of interaction prediction in particular, there is a significant increase of the AUROC from 0.77 (dMaSIF) to 0.88 (our model). As the Orthogonal dataset includes more diversity, we conclude that our model can generalize better to a wide range of protein–protein pairs. Note that we used the default configuration (three layers, patch size 12 Å) for the dMaSIF model, and it might be possible to improve its results on this dataset through hyperparameter tuning.

### 3.3. Evaluation on Ternary Complex Data

We now turn to ternary complex data to evaluate the usefulness of our model for the development of new drugs for targeted protein degradation. In particular, we are interested in recovering the protein–protein interactions involved in ternary complexes using our model. A high recovery rate indicates that our model captures the essential features underlying ternary complex formation and thus can be used to evaluate previously unconsidered protein pairs to find new potential ternary complexes.

Following [55], we evaluated our trained model on 16 ternary complexes (listed in Table 3). The preprocessing of the proteins involved in the ternary complexes was identical to the general PPI prediction tasks (see Section 2); i.e., it included the construction of molecular surface meshes. We then processed the input data with our model pipeline, generating chemo-geometric features and DGRL embeddings, which were then used to make binary predictions. As this pipeline cannot account for the presence of the degrader molecule in the ternary complex, we ignored them in this analysis.

**Figure 4:**
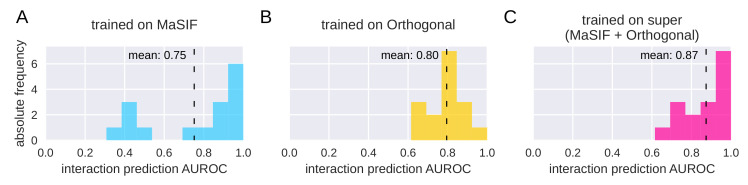
Histograms of AUROC of PPI prediction for the ternary complex dataset defined in Table 3, for models trained on different training sets. (**A**) Results for a model trained on the MaSIF dataset. (**B**) Results for a model trained on the Orthogonal dataset. (**C**) Results for a model trained on the super dataset, i.e., the union of the MaSIF and Orthogonal datasets. The increase of the mean AUROC indicates that the MaSIF dataset lacks diversity of protein structures; thus, it is not sufficient for the development of models in the context of targeted protein degradation. Adding the structures of the Orthogonal dataset leads to a strong improvement. See Table A5, Table A6 and Table A7 in Appendix B for the underlying results on each ternary complex, and Table A8 and Table A9 in Appendix B for exemplary confusion matrices using a threshold of 0.5.

We evaluated the interaction prediction AUROC on the ternary complex data for our model, which was trained on different datasets (Figure 4). When trained on the MaSIF dataset, the mean AUROC is 0.75, and the histogram of AUROCs is bimodal, with a few very low AUROC scores (Figure 4A). From this, we conclude that some of the proteins within the ternary complex dataset are very dissimilar to the data contained in the MaSIF dataset (i.e., they are out of distribution). Thus, the predictions on these proteins are very poor. We next evaluated the Orthogonal dataset (Figure 4B). Here, the mean AUROC has improved to 0.80, and there are no AUROC scores < 0.6. We thus conclude that the Orthogonal dataset with its larger diversity (see above) better reflects the proteins involved in the ternary complexes, and thus, this dataset is more useful for models involved in the prediction of ternary complexes.

When comparing Figure 4A,B, we find that though the mean has improved, the number of very high AUROC scores has decreased. We hypothesized that part of the MaSIF dataset is, in fact, very useful for the prediction of PPIs on the ternary complex data and that training jointly on both datasets could improve the predictions. We therefore re-trained our model on this “super”-dataset (MaSIF + Orthogonal), consisting of 8151 protein pairs for training and 2411 for evaluation. As expected, this leads to an even higher mean AUROC on the ternary complex data (0.87 instead of 0.80 and 0.75 individually, as shown in Figure 4). This provides further evidence that for the prediction of PPIs within a ternary complex, models require datasets that include the largest available diversity.

**Figure 5:**
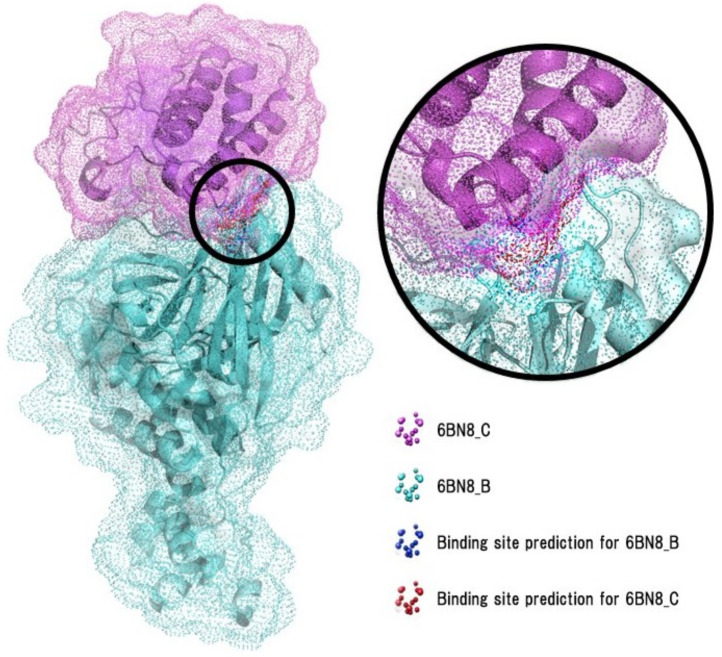
Biochemical representation of predictions for the CRBN–BRD4 complex without the dBET55 degrader (PDB ID: 6BN8). Surface point clouds are superimposed to the original PDB structure. Blue and red points indicate interacting points prediction for chains B and C, respectively (AUROC: 0.987, inference performed using the model trained on the “super” dataset).

From the high accuracy of this final model, we conclude that the combination of the proposed geometric deep learning architecture paired with the novel dataset can accurately determine whether interactions between the two proteins involved in the ternary complexes will take place. To demonstrate the ability of the algorithm to detect the optimal binding site, Figure 5 shows the surface point clouds that are hypothesized to determine the binding sites of both chains of the 6BN8 ternary complex (without a degrader). We find that the algorithm can properly detect both the binding sites of each protein and the main interaction site between the two proteins.

Overall, these results suggest that PPI prediction with DGRL is a promising tool for targeted protein degradation, and it can be used, e.g., as a filtering mechanism to select possible protein-of-interest and E3 ligase pairs, which will, given a suitable degrader molecule, form a ternary complex.

## 4. Discussion

The machine learning model presented in this work used a novel DGRL pipeline based on surfaces meshes and was shown to be capable of predicting binding sites and interactions between proteins with high accuracy. We have furthermore presented a new, complex dataset that highlights the generalization capabilities of our model, demonstrating an improvement over previous work. Toward our primary goal of using protein–protein interaction (PPI) prediction for targeted protein degradation (TPD), we have demonstrated that our model can accurately predict the PPIs on the presently available ternary complex data.

### 4.1. Related Work

We showed that our model reaches results comparable to the MaSIF [30] and dMaSIF [26] models on the MaSIF dataset [30]. While dMaSIF, the current state-of-the-art model for PPI prediction based on 3D structural information, reaches a higher AUROC for binding site prediction, our model performed better than dMaSIF on the task of predicting interactions between pairs of proteins. For our goal of using PPI prediction for TPD, for which a model should determine pairings of a target protein and an E3 ligase, the latter task can be considered more important, and thus, our results constitute an improvement over the dMaSIF model. Furthermore, although our model design differs from dMaSIF in several important ways (e.g., we process explicitly computed surface meshes), inference in our model is still rapid (a few seconds per pass on a Tesla V100 machine), thus making it possible to screen many possible candidate pairs.

A variety of models for PPI prediction have been recently proposed, with some also using the MaSIF dataset as a benchmark. The model presented in [57] achieved AUROC scores of ≤0.71 on this dataset, which is less than both dMaSIF and our model. The model presented in [58] was based on segmentation, which is problematic if only small portions of the protein surfaces overlap. Therefore, the authors used a modified version of the MaSIF dataset, rendering the results incomparable to ours [58]. While these models are based on the prediction of PPIs based on 3D structural information (template-based approaches), several models (e.g., [24,59,60,61]) follow the template-free paradigm and aim to predict PPIs only based on sequence information, with final results which are hard to compare to structure-based models as different datasets and evaluation metrics are used. In some cases, these models employ similar graph-based techniques as our model (e.g., [59], in which PPI networks are modeled using a graph variational autoencoder), highlighting their usefulness. We note that since the prediction of protein structure from sequence information has approached experimental accuracy [62], we expect that these different approaches will converge in the future. This may result in models that use the best of both worlds by first predicting the 3D structure and then incorporating the various techniques (e.g., DGRL or 3D convolutions [63]) used to predict PPIs from structural information.

### 4.2. The Importance of Diverse Datasets for PPI Prediction

It is well known in machine learning that adequate datasets are required for models to be useful. In particular, the dataset used for training a model should cover all the relevant diversity of possible inputs. If this is not the case, then the model will not perform well on new, unseen data (i.e., it will not generalize well). This highlights the benefit of our new, more diverse Orthogonal dataset: as it includes protein pairs with significant differences to those within the MaSIF dataset; testing models on this dataset results in a more accurate estimate of the expected performance of a model on real-world data. Thus, the significantly higher performance of our model on this dataset suggests that it better captures the relevant features of diverse proteins than the dMaSIF model.

This is also corroborated by the analysis of our model on the ternary complex data, which revealed that adding the data from the Orthogonal dataset to the training data dramatically increases the prediction results. This shows that our new dataset is particularly valuable for PPI prediction in this context. We note that the dataset we employed here only contained positive examples of proteins involved in the ternary complex. For a better estimate of how well models can predict PPIs within ternary complexes, enhanced datasets with decoys (i.e., protein pairs with no interactions) as well as more data (as it becomes available) should be used in future works.

### 4.3. Using PPI Prediction for Targeted Protein Degradation

As we have shown, training on a dataset that includes real-world diversity is crucial for machine learning models to be used in the context of targeted protein degradation. Our results suggest using a PPI prediction model as part of (e.g.,) a filtering step in a preliminary screening process, where potentially interacting POIs and E3 ligases are identified for subsequent in-depth analysis. Such an approach can utilize the benefits of machine learning-driven methods to limit the number of potential candidates which need to be evaluated in computationally costly docking simulations or in time- and resource-intensive lab experiments.

One drawback of the current approach is that the presence of the degrader molecule is not taken into account. As there are three separate entities underlying a ternary complex, the PPIs there do not represent classical binary PPIs. Neglecting the degrader nevertheless constitutes a reasonable approximation when using PPI prediction as a proxy to predict potential ternary complexes, as the PPIs between the POI and the E3 ligase are an essential factor underlying ternary complex stability (Figure 1C; see, e.g., [18,64]). For a ternary complex to be formed, positive cooperativity (i.e., PPIs) between the proteins is required; otherwise, only binary complexes (i.e., the degrader binding only to individual proteins) are formed. The approach presented here is based on the assumption that the factors underlying PPIs within ternary complexes are essential the same as in classical binary PPIs; e.g., portions of the protein surface which do not interact favorably with the solvent (water) are more likely to allow PPIs. However, future work should also explicitly take into account the presence of the degrader molecule, in particular, the physical constraints it imposes on the interaction of the two proteins. Recent machine learning approaches such as PhysNet [65] or DimeNet [66] would be well-suited to endow models such as dMaSIF or the one proposed in this work with the required spatial knowledge for generating (if possible) a degrader conformation. Another aspect that should be taken into account is the interactions between the degrader molecule and the proteins, for which the prediction of possible binding sites (as provided by our model or dMaSIF) may be useful.

Apart from targeted protein degradation with bifunctional degraders, there are also molecular glues [67], for which a monovalent small molecule alters the conformation of a protein in a way that enables PPIs. In this case, if the modified structures are available, PPI prediction (without taking into account the degrader) is enough to predict the efficacy of the degradation, and models such as the one presented in this work should allow predicting potential POI–E3 ligase pairs.

### 4.4. PPI Prediction and Complementary Experimental Methods

While computational methods have proven successful for the prediction of protein interactions, they can be complemented by experimental techniques which provide additional structural information on the exact nature of the aggregation and potential conformers. Examples include protein microarrays [68], coimmunoprecipitation [69], protein fragmentation complementation [70], Förster resonance energy transfer (FRET) [71], bioluminescence resonance energy transfer (BRET) [72], fluorescence cross-correlation spectroscopy (FCCS) [73], magnetic tweezers [74], and atomic force microscopy-based force spectroscopy (AFM-FS) [75]). For instance, atomic force microscopy (AFM) can reveal the formation of protein molecular structures as the human mitochondrial apoptosis-inducing factor (hAIF) [76], which are involved in the degradosome formation [77]. Thus, combining multiple methodological approaches has the potential to lead to more accurate results.

## 5. Conclusions

We have demonstrated a novel DGRL pipeline based on surfaces meshes that has proven to be capable of predicting binding sites with high accuracy and beating the state-of-the-art performance using AUROC scores on the established MaSIF dataset for interactions between proteins (Table 2). Furthermore we tested generalization capabilities of our model by constructing a more diverse dataset coined Orthogonal dataset (Table 1). Using this dataset, we have proven that for both tasks of predicting the binding sites and predicting interactions between proteins, we can achieve better results than another state-of-the-art DGRL pipeline (Table 2).

To further evaluate these generalization capabilities, we used our model to predict the PPIs relevant for targeted protein degradation, where we demonstrate high accuracy of our model for PPI prediction on the available ternary complex data (Table A7).

All of the results suggest that our DGRL pipeline can be an informative tool for screening protein pairs while developing new drugs for targeted protein degradation.

## Figures and Tables

**Table 1:** Comparison between the MaSIF and Orthogonal datasets. Columns show (from left to right) the dataset name, the considered task (prediction of binding sites or protein–protein interactions), the subset (training or test set), the number of PDB IDs within the datasets, the average resolution of the PDB IDs within the datasets in Å, the average molecular weight per deposited model, the average total number of polymer residues per deposited model, the average polymer entity sequence length, and the average molecular entity weight.

Dataset	Task	Subset	PDB IDs	Resolution (Å)	Mol. w. per Model (kDa)	Tot. Polymer Residues per Model	Polymer Entity seq. Length	Mol. Entity w. (kDa)
MaSIF	binding site	train.	2809	2.27	99.08	873.58	220.32	24.7
		test	368	2.35	142.42	1270.48	343.61	38.21
	interactions	train.	4833	2.35	117.92	1043.45	264.32	29.67
		test	970	2.28	99.31	876.54	235.81	29.9
Orthogonal	binding site	train.	2373	1.75	66.07	547.07	246.82	27.94
		test	1111	1.81	105.54	880.11	297.68	33.81
	interactions	train.	3201	1.77	80.86	657.90	214.36	26.82
		test	1431	1.74	65.06	566.08	249.67	28.21

**Table 2:** Evaluation of PPI prediction models. The models’ AUROC scores were evaluated on the test set of the MaSIF dataset and on the test set of our new Orthogonal dataset for the two tasks of binding site and interaction prediction Table 1. There we made sure that the ratio of the test data to training data is very high, hence evaluating test set is likely to be a more accurate and reliable indicator of the generalization capabilities. While our model does not significantly improve the results of dMaSIF [26] on the MaSIF dataset (with better results for interaction prediction but inferior results for binding site prediction), it significantly outperforms dMaSIF on the new Orthogonal dataset, which better captures the diversity of possible protein binding pairs. The results that we obtained using dMaSIF model used the default configuration (three layers, patch size 12 Å) of the model. No modifications of the dMaSIF code or model were made. For exemplary confusion matrices using a threshold of 0.5, see Table A1, Table A2, Table A3 and Table A4 in Appendix A. Additionally, in the same Appendix, we detailed the analysis on how much the accuracy of each model is above the corresponding random accuracy, as described in [53,54]. Finally, bolded values in this table are the superior AUROC (area under the receiver operating characteristic curve) values. These AUROC values are summarized predictions across different surface patches, explanation on calculating the same can be found in Section 2.

Dataset	Task	MaSIF [30]	dMaSIF [26]	Ours
MaSIF	binding site	0.85	**0.87**	0.82
	interactions	0.81	0.82	**0.88**
Orthogonal	binding site	-	0.77	**0.79**
	interactions	-	0.77	**0.88**

**Table 3:** Ternary complex dataset. In addition to the unique PDB identifiers and two chains we select for prediction, we also quote the name of E3 ligases and targets/POI (and domain of binding if applicable) [55,56]. Note that for the PDB identifier 6W8I, three distinct pairs of chains are considered, which define BTK bound to the BIR3 domain of cIAP1.

PDB ID	Chains	E3 ligase	Target
5T35	A, D	VHL	BRD4 BD2
6BN7	B, C	CRBN	BRD4 BD1
6BN8	B, C	CRBN	BRD4 BD1
6BN9	B, C	CRBN	BRD4 BD1
6BNB	B, C	CRBN	BRD4 BD1
6BOY	B, C	CRBN	BRD4 BD1
6HAX	A, B	VHL	SMARCA2
6HAY	E, F	VHL	SMARCA2
6HR2	E, F	VHL	SMARCA4
6SIS	E, H	VHL	BRD4 BD2
6W7O	B, D	cIAP1-BIR3	BTK
6W8I	A, D ∣ B, E ∣ C, F	cIAP1-BIR3	BTK
6ZHC	AD	VHL	Bcl-xL
7KHH	CD	VHL	BRD4 BD1

## Data Availability

The MaSIF dataset is publicly available at https://zenodo.org/record/2625420, accessed on 9 May 2022. The new Orthogonal dataset is publicly available at https://ppi-orthogonal-data.s3.eu-central-1.amazonaws.com/orthogonal_dataset.zip, accessed on 9 May 2022.

## Abbreviations

The following abbreviations are used in this manuscript:

PPI	protein–protein interaction
CNS	central nervous system
POI	protein of interest
PIC	proximity-inducing compound
TPD	targeted protein degradation
CRBN	Cereblon
VHL	Von Hippel–Lindau tumor suppressor
DGRL	deep graph representation learning
PDB	Protein Data Bank
*k*-NN	*k* nearest neighbors
MLP	multilayer perceptron
AUROC	area under the receiver operating characteristic curve

## Appendix A. Confusion Matrices on MaSIF and Orthogonal Datasets

**Table A1:** Confusion matrix for binding site prediction with our model on the MaSIF dataset (achieving an AUROC of 0.82, see Table 2). True/false positives and true/false negatives are averaged across multiple different surfaces patches (see Section 2). The threshold was set to 0.5. *Accuracy* = (TP + TN)/*N* = 0.773, $Accuracy_{random}$ = ((TP + FN)(TP + FP) + TN + FP)(TN + FN)/($N^{2}$) = 0.505, $Real_{Accuracy}$ = $Accuracy-Accuracy_{random}$ = 0.268 (26.8%) ([53,54]).

		Predicted	
		interaction	no interaction
Actual	interaction	3273 (32.7%)	1380 (13.8%)
	no interaction	882 (8.8%)	4465 (44.6%)

**Table A2:** Confusion matrix for interaction prediction with our model on the MaSIF dataset (achieving an AUROC of 0.88, see Table 2). Details as in Table A1. *Accuracy* = (TP + TN)/*N* = 0.797, $Accuracy_{random}$= ((TP + FN)(TP + FP) + TN + FP)(TN + FN)/($N^{2}$) = 0.501, $Real_{Accuracy}=Accuracy-Accuracy_{random}$ = 0.296 (29.6%) ([53,54]).

		Predicted	
		interaction	no interaction
Actual	interaction	3418 (34.2%)	1314 (13.1%)
	no interaction	712 (7.1%)	4556 (45.5%)

**Table A3:** Confusion matrix for interaction prediction with our model on the Orthogonal dataset (achieving an AUROC of 0.79, see Table 2). Details as in Table A1. *Accuracy* = (TP + TN)/*N* = 0.736, $Accuracy_{random}$ = ((TP + FN)(TP + FP) + TN + FP)(TN + FN)/($N^{2}$) = 0.505, $Real_{Accuracy}=Accuracy-Accuracy_{random}$ = 0.231 (23.14%) ([53,54]).

		Predicted	
		binding site	no binding site
Actual	binding site	3210 (32.1%)	1714 (17.1%)
	no binding site	926 (9.2%)	4150 (41.5%)

**Table A4:** Confusion matrix for interaction prediction with our model on the Orthogonal dataset (achieving an AUROC of 0.88, see Table 2). Details as in Table A1. *Accuracy* = (TP + TN)/*N* = 0.797, $Accuracy_{random}$ = ((TP + FN)(TP + FP) + TN + FP)(TN + FN)/($N^{2}$) = 0.505, $Real_{Accuracy}=Accuracy-Accuracy_{random}$ = 0.292 (29.2%) ([53,54]).

		Predicted	
		interaction	no interaction
Actual	interaction	3503 (35.1%)	914 (9.1%)
	no interaction	1107 (11.0%)	4476 (44.7%)

## Appendix B. Prediction Results for the Known Ternary Complexes

**Table A5:** AUROC scores for PPI prediction of our model trained on the MaSIF dataset (see Figure 4, panel A).

PDB ID	Chains	PPIs AUROC
5T35	A, D	0.40
6BN7	B, C	0.93
6BN8	B, C	0.95
6BN9	B, C	0.95
6BNB	B, C	0.86
6BOY	B, C	0.95
6HAX	A, B	0.46
6HAY	E, F	0.45
6HR2	E, F	0.51
6SIS	E, H	0.38
6W7O	B, D	0.72
6W8I	A, D	0.89
6W8I	B, E	0.80
6W8I	C, F	0.81
6ZHC	A, D	0.95
7KHH	C, D	0.93

**Table A6:** AUROC scores for PPI prediction of our model trained on the orthogonal dataset (see Figure 4, panel B).

PDB ID	Chains	PPI AUROC
5T35	A, D	0.76
6BN7	B, C	0.78
6BN8	B, C	0.86
6BN9	B, C	0.78
6BNB	B, C	0.83
6BOY	B, C	0.81
6HAX	A, B	0.67
6HAY	E, F	0.66
6HR2	E, F	0.67
6SIS	E, H	0.71
6W7O	B, D	0.84
6W8I	A, D	0.92
6W8I	B, E	0.96
6W8I	C, F	0.77
6ZHC	A, D	0.91
7KHH	C, D	0.78

**Table A7:** AUROC scores for PPI prediction of our model trained on the super-dataset (see Figure 4, panel C). See Table A8 and Table A9 for exemplary confusion matrices for the complexes 6BN8 and 6HR2, respectively.

PDB ID	Chains	PPI AUROC
5T35	A, D	0.76
6BN7	B, C	0.98
6BN8	B, C	0.99
6BN9	B, C	0.97
6BNB	B, C	0.94
6BOY	B, C	0.98
6HAX	A, B	0.84
6HAY	E, F	0.74
6HR2	E, F	0.69
6SIS	E, H	0.72
6W7O	B, D	0.81
6W8I	A, D	0.86
6W8I	B, E	0.87
6W8I	C, F	0.91
6ZHC	A, D	0.97
7KHH	C, D	0.97

**Table A8:** Exemplary confusion matrix for interaction prediction with our model the ternary complex 6BN8 (one of the best scoring complexes with an AUROC of 0.99, see Table A7). True/false positives and true/false negatives are averaged across multiple different surfaces patches (see Section 2). The threshold was set to 0.5.

		Predicted	
		interaction	no interaction
Actual	interaction	4423 (44.2%)	112 (1.1%)
	no interaction	92(1.0%)	5373(53.7%)

**Table A9:** Exemplary confusion matrix for interaction prediction with our model the ternary complex 6HR2 (one of the worst scoring complexes with an AUROC of 0.69, see Table A7). Details as in Table A8.

		Predicted	
		interaction	no interaction
Actual	interaction	2943 (29.4%)	2382 (27.8%)
	no interaction	1108 (11.1%)	3567 (35.6%)
